# A La Carte Seed Harvesting: *Messor barbarus* Ants Select Durum Wheat Genotypes

**DOI:** 10.1002/ece3.72251

**Published:** 2025-10-03

**Authors:** Clément Plessis, Aline Rocher, Frédéric Compan, Jonathan Romiguier, Jacques David, Hélène Fréville

**Affiliations:** ^1^ AGAP Univ. Montpellier, CIRAD, INRAE, Institut Agro Montpellier France; ^2^ DIASCOPE Univ. Montpellier, INRAE, Institut Agro Montpellier France; ^3^ ISEM Univ. Montpellier, CNRS, IRD Montpellier France

**Keywords:** cafeteria experiment, crop evolution, evolutionary ecology, genome‐wide association study, harvester ant preference, plant genetic diversity, plant–ant interactions, seed foraging

## Abstract

Biotic interactions between plants and insects can drive key evolutionary processes. In Mediterranean agroecosystems, the harvester ants *Messor barbarus* (Hymenoptera: Formicidae) frequently collect seeds, including those of cultivated cereals. Yet their potential role in shaping crop traits remains poorly understood. This study investigates whether harvester ant seed predation is driven by genetic and phenotypic variation in durum wheat ( *Triticum turgidum* ssp. *durum* ), a major Mediterranean crop derived from wild emmer ( *T. turgidum* ssp. *dicoccoides*). Using a panel of 180 genetically diverse durum wheat inbred lines grown in a field experiment, we visually recorded spike predation and performed a genome‐wide association study (GWAS) using SNP markers to assess the genetic architecture of susceptibility to seed predation by *M. barbarus* . We identified a significant quantitative trait loci (QTL) on chromosome 2A explaining 21% of the variation in predation rate. This region contains a 3.6 Mb chromosomal inversion and 46 candidate genes, including a MYB transcription factor potentially involved in regulating cuticle and chemical traits. To validate these genetic findings, we conducted a cafeteria experiment with 208 spikes from 26 genotypes, placed at the entrances of eight ant nests. Ants preferentially removed spikes from genotypes carrying the allele identified in the GWAS. Additionally, shorter spikes were more likely to be harvested. However, unlike previous studies on wild plants, seed morphology and protein content did not significantly affect ant preference. *Synthesis*. Our results demonstrate that *M. barbarus* exhibits genotype‐specific preferences in durum wheat, associated with a major QTL, and is influenced by spike traits. This study provides the first evidence of ant‐mediated selective pressure in a cereal crop and opens new perspectives on plant–insect dynamics in agroecosystems and the role of plant–insect interactions in the evolutionary history of crop species.

## Graphical abstract

Our study shows that the harvester ant *Messor barbarus* exhibits genotype‐specific preferences for durum wheat, linked to a major QTL on chromosome 2A. Ant predation is also influenced by spike size, suggesting ant‐mediated selective pressure on crop traits. Our results may provide the first evidence of evolutionary interactions between harvester ants and crops in Mediterranean agroecosystems.

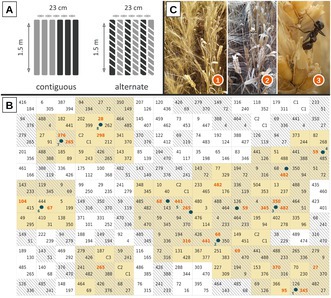

## 1 Introduction

Interactions between plants and insects are major drivers of evolution, fostering the emergence of complex adaptive traits and shaping ecosystems (Ehrlich and Raven 1964). From the plant's perspective, antagonistic relationships, such as insect herbivory, can negatively impact plant fitness (Ehrlich and Raven 1964; Karban 2020) and drive the evolution of defense mechanisms, such as trichomes or tissue bitterness (Mauricio and Rausher 1997; Rashid War et al. 2018). Conversely, mutualistic interactions like pollination enable plants to reproduce while insects benefit from nectar (Kevan and Baker 1983; Bronstein et al. 2006). Seed predation, the partial or total consumption of seeds also called granivory, can be viewed as either a mutualistic or antagonistic interaction, depending on the balance between its costs and benefits for the plant species. Seed predators can exert strong selective pressures on plant populations, driving the evolution of traits such as seed size, dispersal mechanisms, and defensive traits (Janzen 1971; Hulme 1998).

Harvester ants specialize in collecting seeds, transporting them to granaries for partial or complete consumption, playing a role of both dispersers and predators (Retana et al. 2004). In response, many plant species have evolved traits that reduce seed harvest or consumption on the plant. For instance, tussock grass ( *Stipa tenacissima* ) produces elongated diaspores that self‐bury in the soil (Schöning et al. 2004), while other species have mucilaginous seeds that adhere to surfaces upon contact with water, hindering ant collection (Pan et al. 2021). Conversely, some plants have taken advantage of ant foraging behavior for seed dispersal. This is the case with myrmecochory, where nutrient‐rich elaiosomes encourage ants to transport seeds without consuming them (Anjos et al. 2020; Karnish 2024). However, not all plant species exhibit such adaptations: some experience substantial predation but may nonetheless benefit from it when ants inadvertently drop seeds along their foraging paths or leave them uneaten in their granaries (Retana et al. 2004; Arnan et al. 2012). In the most adverse scenarios for the plant, ants consume nearly all seeds, exerting strong selection pressure in the vicinity of ant nests (White and Robertson 2009; Baraibar, Carrión, et al. 2011). Coevolution syndromes have been described for various ant‐plant interacting species in natural ecosystems (Ehrlich and Raven 1964; McKey et al. 1999). However, despite the widespread presence of ants in agrosystems, ant–crop coevolution remains largely underexplored.

In Europe, harvester ants of the *Messor* genus mainly live in semi‐arid climates around the Mediterranean Sea, where they are found in both natural ecosystems and agrosystems. In these regions, durum wheat ( *Triticum turgidum* ssp. *durum* ), a key staple crop cultivated for thousands of years, occasionally experiences seed predation by harvester ants (Baraibar, Ledesma, et al. 2011; Lev‐Yadun et al. 2015). Its wild progenitor, *Triticum turgidum* ssp. *dicoccoides* occurring in the Fertile Crescent, is also predated by harvester ants, which disarticulate spikelets and carry the seeds to their nest (Lev‐Yadun et al. 2015). As durum wheat, *Messor* harvester ants are estimated to have originated and diversified near the Fertile Crescent, in the Indo‐Iranian bioregion (Juvé et al. 2025). There is ample evidence that domestication and plant breeding have shifted seed morphological and dispersal traits (Gepts 2003; Milla 2023). For instance, cereal seed crops such as durum wheat have evolved larger seed sizes, and nonshattering—a widely recognized key domestication trait—prevents natural dispersal. However, the extent to which harvester ants have imposed additional selective pressures on these seed traits remains poorly understood. In natural ecosystems, ants tend to avoid seeds with excessively large dispersal structures but generally target the longest and heaviest seeds among those they are able to transport (Azcárate et al. 2005; Heredia and Detrain 2005; Azcárate and Peco 2006). Elucidating the genetic basis of plant traits underlying ant preferences would advance our understanding of the evolutionary processes that shape harvester ant–crop interactions. To date, however, no study has explored how intraspecific genetic variation in crops affects variation in seed predation by harvester ants.

Building on observations from a field experiment originally designed to assess the impact of durum wheat varietal mixtures on fungal diseases, we investigated the phenotypic and genetic determinants of seed predation by the harvester ant *Messor barbarus* across durum wheat genotypes exhibiting high phenotypic and genetic diversity. First, we performed a genome‐wide association study (GWAS) using single nucleotide polymorphisms (SNPs) to uncover the genetic basis of harvester ant predation on durum wheat. Then, based on the identified genomic regions, we examined the functions of candidate genes potentially involved in ant predation. Finally, we performed a cafeteria experiment to validate our genetic findings and investigate how spike and seed traits influence ant preferences. To do so, we estimated the heritability of wheat on ant preference and tested whether ant preference is partly genetically determined in wheat. We then analyzed the selective pressure exerted by ants on spike and seed traits, such as morphological characteristics and protein content, to better understand the selective pressures at play.

## 2 Materials and Methods

### 2.1 Plant Material

**FIGURE 1:**
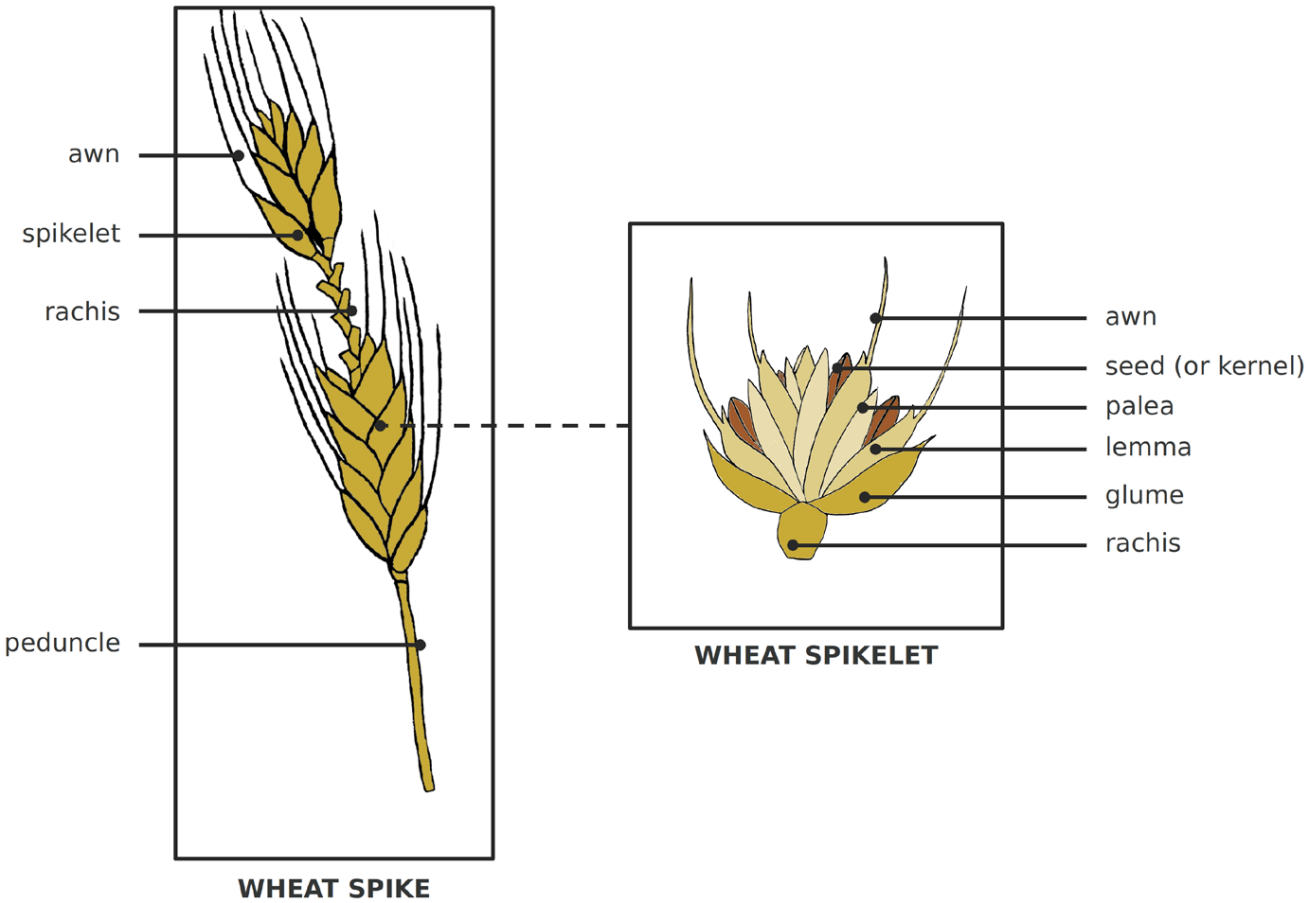
Illustrations of a mature modern wheat spike and a spikelet. The spikelet is the dispersal unit in the wild wheat. During domestication, humans selected for free naked seeds, free threshing, and rachis nonbrittleness. A spikelet contains, on average, 2 to 5 seeds in durum wheat.

We used 180 genotypes corresponding to inbred lines derived from the Evolutionary PreBreeding durum wheat Population (EPO), a genetically diverse, outcrossed wheat tetraploid population founded in 1997 at INRAE Montpellier, France (David et al. 2014). The 180 genotypes derived from the evolution of a large initial set of accessions from *Triticum turgidum* ssp. including wild ( *Triticum turgidum* spp. *dicoccoides*), primitive ( *Triticum turgidum* spp. *diccococum*), and modern subspecies of durum wheat ( *Triticum turgidum* spp. *durum*), which intercrossed during 17 generations thanks to the segregation of a male sterility nuclear gene in 2009. The population evolved in response to local environmental pressures as well as human selection for agronomic phenotypes such as nonbrittle rachis to prevent spikelet dispersal and naked seeds (Figure 1). Spikes from the 17th generation were collected and underwent two successive generations of selfing by single‐seed descent to produce the 180 inbred lines used in this study. These lines exhibit high genetic diversity and high phenotypic variability for both above‐ and belowground traits and represent ideal material for genome‐wide association studies (Ballini et al. 2020; Colombo et al. 2022). All lines were genotyped using the TaBW280K high‐throughput genotyping array (Rimbert et al. 2018), which provides high‐density SNP markers. Polymorphic SNPs are biallelic and distributed across all 14 chromosomes of durum wheat. Physical positions of SNPs were annotated based on Ensembl Svevo.v1 variant information.

### 2.2 Field Observations

#### 2.2.1 Experimental Design

**FIGURE 2:**
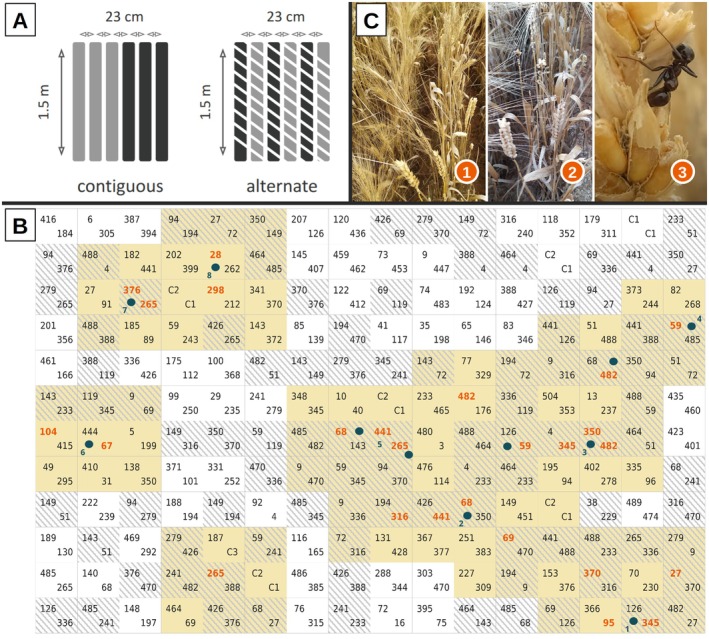
(A) The two sowing conditions within the field experiment where light gray and dark gray represent respectively one genotype and hatching refer to alternate lines. (B) The areas of the experimental field prospected by *Messor barbarus* . Each box represents a 6‐row plot of 1.5 m in length, containing two of the 180 durum wheat genotypes and three commercial varieties (C1, C2, C3). Hatching indicates alternate lines plots, otherwise a contiguous one. The foraging area is in yellow where each plot had at least one neighbor on which *Messor barbarus* had been observed (orange number) at least once. Only these four areas were considered for the GWAS. Blue dots indicate nest main entries and blue numbers the eight cafeterias places (see below). (C) 1: A nonpredated genotype on the left and a predated one on the right in an alternate lines plot, 2: A predated genotype with score 4, 3: A *Messor barbarus* worker disarticulating spikelets.

We sowed the experiment at Mauguio, southern France (INRAE–UE DIASCOPE—43°37′10.7″N 3°59′00.22033″E) on November 13th, 2023, in an area free of pesticides since 2008. The experiment consisted of 192 randomized nursery plots of six 1.5 m long rows, with 23 cm between rows. We sowed each plot with two genotypes and two spatial arrangements (Figure 2A): 95 plots in which each of the 180 EPO genotypes and three commercial varieties (each represented once) were sown on three contiguous lines; and 97 plots in binary mixtures with alternate lines using 32 genotypes randomly drawn among the 180 EPO genotypes (Figure 2B). We originally designed this experiment to test the effect of varietal mixture on fungal disease. This design allows testing the performance of a genotype when it is surrounded by itself (for 180 genotypes) or by another genotype (for 32 genotypes).

#### 2.2.2 Foraging Area and Predation Levels

**FIGURE A1:**
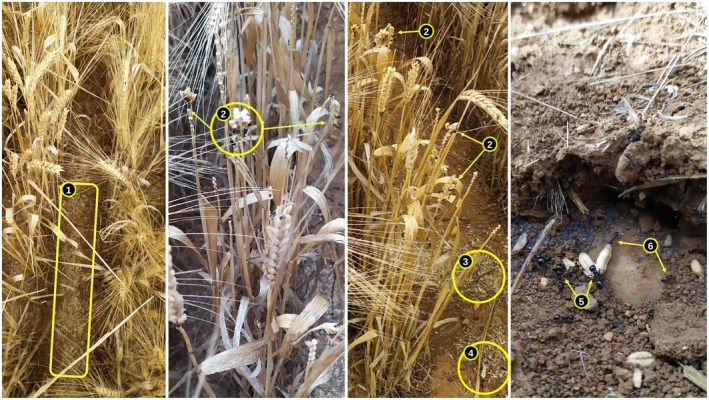
In field observations of wheat predation: (1) awns and glumes at the foot of a line attacked by ants (2) ants disarticulating spikelets on plants (3) pile of waste at an ant nest entrance (4) ants collecting seed on spike close to a pile of waste (5) *Messor barbarus* “Major” workers (6) *Messor barbarus* “Minor” workers.

During field notations on June 6th, 2024, we detected the presence of harvester ants on wheat spikes (Figure 2C and Appendix 1: Figure A1). The ant species was further unequivocally identified as *Messor barbarus* . For each genotype in each plot, we assessed a visual Harvest Score (*HS*) on a scale of 0 to 4, with 0 corresponding to no observed ant and no visual sign of spike predation, 1 to the presence of ants but no evidence of predation, 2 to ants collecting seeds and predated spikes, 3 to at least one entire predated row, and 4 to multiple predated rows. As we did not observe ants over the whole area of the experiment, we defined foraging areas by gathering all plots with at least one neighboring plot with a score of at least 1, in any direction, resulting in four noncontiguous foraging areas (Figure 2B). The whole foraging area contained 97 plots representing a total of 95 EPO genotypes being represented twice on average. On June 6th, 21st, and 28th, we recorded ant activity and the presence of ant nest entrances, resulting in the identification of 11 active ant nest entrances. We located ant nests by following trails until detecting a pile of waste at ant nest entrances, which was predominantly composed of wheat rachis, glumes, lemma, and palea (Appendix 1: Figure A1).

#### 2.2.3 Genome‐Wide Association Between Markers and Predation Levels

To identify which plant genomic regions might be linked to ant predation, we conducted a Genome‐Wide Association Study (GWAS) on the harvest score of the 95 EPO genotypes present in the foraging area. This dataset comprised 193 values of *HS* (Figure 2B). We used the 38,983 SNP surpassing the minor allele frequency (MAF) threshold of 10% to have at least nine individuals carrying the alternative allele. In a first step, because many genotypes appeared only once (63 out of 95), we used Best Linear Unbiased Predictors (BLUPs), a statistical method used to estimate genetic values related to one or several phenotypes when data is limited or unbalanced. BLUPs were generated using the “sommer” package (Covarrubias‐Pazaran 2016) with a VanRaden kinship matrix (VanRaden 2008) computed from the genotyping data.

In a second step, we assessed the effect of each SNP marker on genetic values of *HS*, using the following model:

y=Xβ+Zu+ϵ

where ***y*** is the vector of 95 BLUP genetic values of *HS*, ***X*** is the incidence matrix of the two alleles at the considered marker and ***β*** the fixed effect, ***Z*** is the incidence matrix for genotypes associated with ***u***, the vector of the polygenic random effect, and ***ϵ*** is the vector of residual effects. The random effects ***u*** are assumed to follow a normal distribution with variance var(***u***) = 2 ***K*** $\sigma_{a}^{2}$ where ***K*** is the kinship matrix and $\sigma_{a}^{2}$ the additive genetic variance.

We fitted this model using the “runSingleTraitGwas” function from the “stagenGWAS” package (Van Rossum and Kruijer 2020). We selected the “multi” option for the parameter “GLSmethod” to gain statistical power and reduce false positives by using kinship associated with each chromosome. Statistical significance of associations was evaluated using a Bonferroni correction threshold. Then, to group all significant linked markers into Quantitative Trait Loci (QTLs), we defined confidence intervals around the most significant SNPs. Intervals included neighboring regions where the statistical values remained above a threshold defined as:

$$-\log_{10}\left(p{\mathrm{val}}_{\text{flank}}\right)\geq -\log_{10}\left(p{\mathrm{val}}_{\text{peak}}\right)–1.5$$

where *p*val_peak_ corresponds to the *p* value of the most significant SNP and *p*val_flank_ the *p* values of the flanking markers located in the same chromosomal region. We verified the physical consistency of the interval by analyzing the linkage disequilibrium (LD) between SNP markers within each QTL region, using the correlation of allelic frequencies between the peak marker and its flanking neighbors (*r* ^2^).

As mentioned above, our panel originates from crosses among *Triticum turgidum* subspecies, including *T*. *t. dicoccoides* (wild emmer wheat) and *T. t. durum* (modern durum wheat). As both subspecies have reference genomes—respectively WEW (Avni et al. 2017, version v1) and Svevo (Maccaferri et al. 2019, version v1)—we analyzed marker positions and compared the genomic structure of QTL regions between subspecies. For each species, we identified the QTLs' physical size by mapping sequences corresponding to significant markers onto the corresponding reference genome using BLAST from the GrainGenes platform (Carollo et al. 2005). Based on the QTLs' position in both subspecies' genomes, we retrieved already identified QTLs and genes from GrainGenes and Ensembl Biomart (Kinsella et al. 2011), respectively. Additionally, to assess the conservation of genomic regions between subspecies, we performed a pairwise alignment of the QTL sequences from wild emmer wheat and modern durum wheat references using “minimap2” v2.26 (Li 2018) and visualized the results with the R package “pafr” v0.0.2 (Winter 2020). We then added SNPs and genes' positions onto the alignment.

### 2.3 Cafeteria Experiment

#### 2.3.1 Experimental Design and Data Collection

**FIGURE 3:**
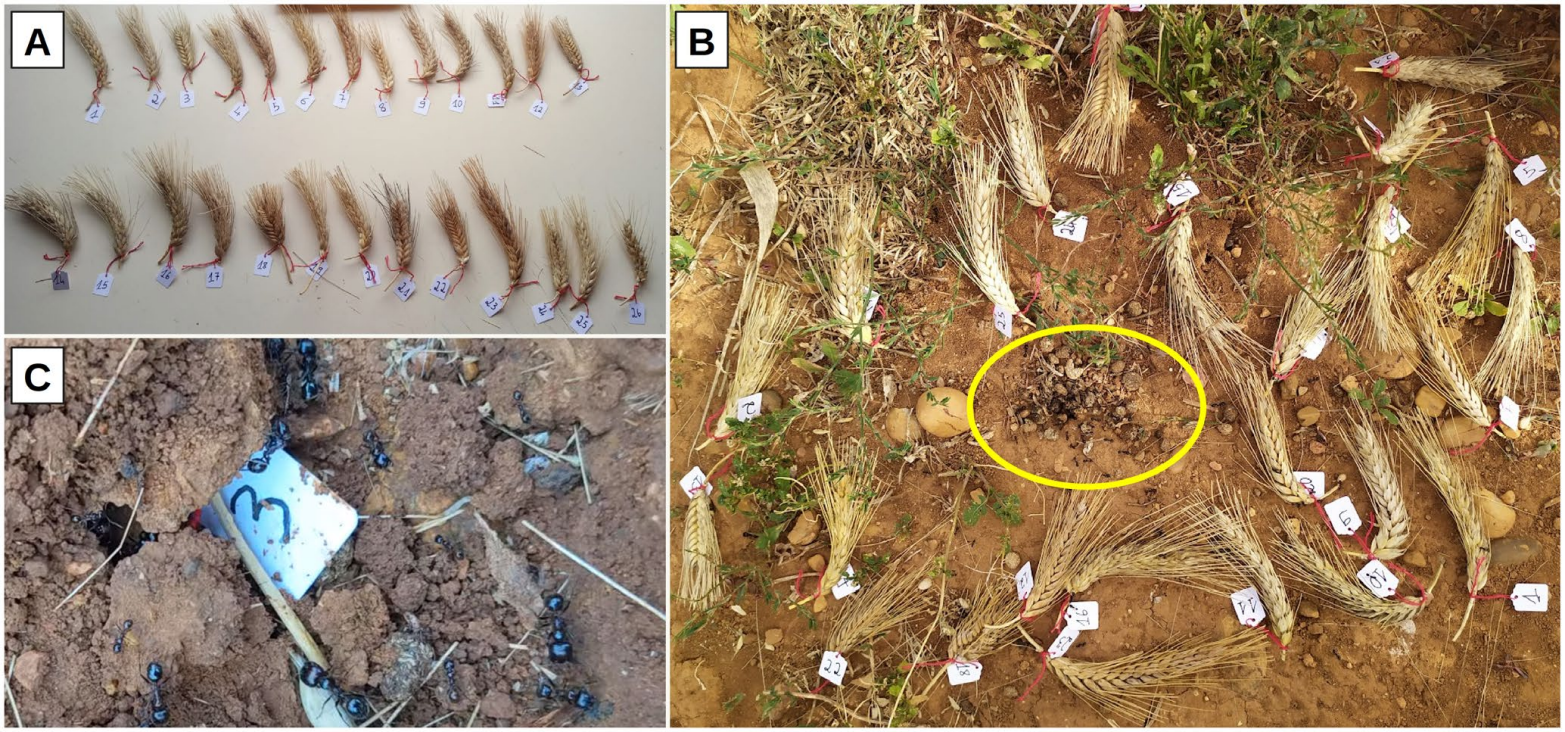
(A) Batch of 26 spikes from the 26 genotypes at *T*_0_; each of them is labeled from 1 to 26; (B) one batch of 26 spikes randomly spread around the ant nest entrance—nest entrance highlighted in yellow; (C) Spike number 3 after 24 h, completely buried into the ant nest.

To assess the ability of ants to discriminate between wheat genotypes and to test whether spike and seed characteristics influence their preferences, we conducted a cafeteria experiment in the observed foraging areas. We used the 17 EPO genotypes that were predated by ants in the field experiment, along with six randomly selected nonpredated EPO genotypes as controls. Additionally, we included two commercial durum wheat varieties (Pescadou and Casteldoux) from our plots, as well as one commercial bread wheat (Apexus) grown adjacent to the experiment, which also experienced ant predation. For each of these 26 genotypes, we collected eight nonpredated spikes in the field experiment, weighed each spike (mass *M*_0_), and measured their length. We then labeled each spike and grouped them to form eight complete replicates, each consisting of a spike for all 26 genotypes (Figure 3A ). In addition, we generated a control dataset to assess seed characteristics of the genotypes we used in the cafeteria experiment. To do so, we collected three unpredated spikes from the field for each of the 26 genotypes grown in the same plot as those used for the cafeteria experiment. The resulting 78 spikes were individually weighed, manually threshed, and their seeds counted. The seed batches were analyzed individually using an automated phenotyping pipeline. This pipeline included an ASD device (near‐infrared spectroscopy), which provided the average spectrum of each seed batch to predict their protein content (Compan et al. 2013). In addition, RGB (red, green, blue) images from each seed were taken to extract key morphological traits, including seed length, width, eccentricity (seed ovality), solidity (continuous shape of the seed), thousand kernel weight, area (the number of pixels within the seed image), and ratios of red, green, and blue colors.

#### 2.3.2 Harvester Ants' Preferences for Spike Characteristics

Each of the eight cafeteria dataset batches was placed on the ground at the eight most active ant nest entrances within the foraging areas at time *T*_0_ (Figure 3B). After 24 h, we collected the spikes of each replicate, weighed them (*M*_24_), and placed each replicate back in the field at another ant nest entrance. After 42 h from *T*_0_, we collected the spikes and weighed them again (*M*_42_). By switching ant nests, we aimed to verify whether the preference observed between 0 and 24 h was reproducible between 24 and 42 h when changing ant nests. If a spike was missing for a given genotype (Figure 3C), its mass was recorded as 0. We then quantified seed predation and ant preferences by calculating various variables based on spike mass data. We conducted the cafeteria experiment in the field before harvesting. Thus, in addition to the spikes provided, the ants still had access to the 180 genotypes present in the field.

First, we defined the absolute mass loss (*ML*) of a spike as a proxy of ant preference. Indeed, a high *ML* value indicates strong ant predation and thus strong preference, while a low *ML* value reflects low predation and thus limited preference. We calculated *ML* per spike after 24 h and 42 h as follows:

$${\mathrm{ML}}_{0–24}=M_{0}–M_{24}$$

$${\mathrm{ML}}_{0–42}=M_{0}–M_{42}$$

Second, to assess the impact of ant predation on spike mass, we calculated the relative mass loss (*RML*) of each spike after 24, 42 h and from 24 to 42 h as follows:

$${\mathrm{RML}}_{0–24}=\left(M_{0}–M_{24}\right)/M_{0}$$

$${\mathrm{RML}}_{0–42}=\left(M_{0}–M_{42}\right)/M_{0}$$

To test whether ants continue to collect seeds from already predated spikes, we also calculated the absolute and relative mass loss from 24 to 42 h as follows:

$${\mathrm{ML}}_{24–42}=M_{24}–M_{42}$$

$${\mathrm{RML}}_{24–42}=\left(M_{24}–M_{42}\right)/M_{24}$$

We then tested the ability of ants to choose spikes depending on wheat genotypes, using a mixed model for each predation variable with the R package lme4 (Bates et al. 2015): we declared genotype as a random effect, considering that the studied genotypes were not the only ones of interest and represented a random sample of a larger population. We estimated broad sense individual heritability (*H* ^2^) for spike mass at *T*_0_ and each predation variable by computing the proportion of phenotypic variation explained by genetic variation as follows:

$$H^{2}=\mathrm{var}\left(G\right)/\left(\mathrm{var}\left(G\right)+\mathrm{var}\left(E\right)\right)$$

with var(*G*) the genetic variance due to the genotype effect, and var(*E*) the residual variance due to all other effects.

We further examined whether spike mass, spike length, and spike density (defined as mass/length) influenced ant preferences using Spearman correlation test on *ML* and *RML*. Finally, we tested whether values of ant preference variables from the cafeteria experiment were consistent with detected QTLs of predation, using a Wilcoxon test to compare them between both allelic states at detected QTLs.

#### 2.3.3 Impact of Ant's Preferences on Remaining Seeds Morphology

To assess the impact of ant preferences on shifts in the seed phenotypic characteristics, we applied the automated phenotyping pipeline (described above) to the seeds that remained within spikes at the end of the cafeteria experiment. For each spike, we obtained a mean seed value of protein content, seed length, seed width, eccentricity, solidity, thousand kernel (seed) weight, size, and ratios of red, green, and blue colors. For each trait and each genotype, phenotypic change was calculated as follows:

$${\mathrm{RV}}_{j}=\left(T_{j}–\mu_{j}\right)/\mu_{j}$$

where *RV*_*j*_ is the seed trait relative value after predation for genotype *j*, *T*_*j*_ is the trait mean value measured for genotype *j* from the remaining seeds after predation, and *μ*_*j*_ is the trait mean value for genotype *j* from the control dataset. Thus, for a given genotype, a positive *RV* value indicates that the mean phenotypic value of the trait increased after 42 h, whereas a negative value indicates a decrease and a null value indicates no change. Then for each trait we tested the relative change with a Wilcoxon test.

Finally, in order to assess whether the changes observed were related to ant predation, we conducted a Spearman correlation test between the predation variables after 42 h (*ML*_0–42_ and *RML*_0–42_) and the seeds' relative values (*RV*).

## 3 Results

### 3.1 Harvest Score Quantitative Trait Loci

#### 3.1.1 Predation by Ants Is Partly Explained by Wheat Genetic Variation

**FIGURE 4:**
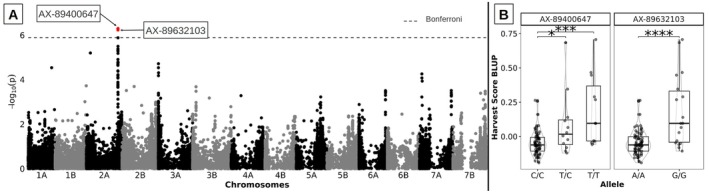
(A) Manhattan plot of the −log_10_ (*p* value) of the 39 K SNPs used in the genome‐wide association study (GWAS) on genetic BLUPs from the visual score of seed predation by *Messor barbarus* on a set of 96 durum wheat genotypes; the two significant SNPs AX‐89400647 and AX‐89632103 are shown in red; (B) Effect of the two significant SNPs AX‐89400647 and AX‐89632103 on predation BLUP's. Note that lines were not completely inbred and were still heterozygous at SNP AX‐89400647.

Out of 38,983 markers, two significant SNPs were identified on chromosome 2A, AX‐89400647 (*p*val = 4.9 × 10^−7^) hereafter referred to as SNP1, and AX‐89632103 (*p*val = 5.8 × 10^−7^) hereafter referred to as SNP2, whose allelic variation explained part of the variation in predation by *M. barbarus* harvester ants (respectively 21% and 18% of the BLUP variance; Figure 4). These markers are located on the distal part of the long arm of chromosome 2A and in strong linkage desequilibrium (*r* ^2^ = 0.79). BLAST‐based mapping of both markers on the GrainGenes platform revealed that the markers are positioned at 687,026,788 bp (SNP1) and 687,643,299 bp (SNP2) on the modern durum wheat reference genome. On the wild emmer wheat reference genome, the same markers are respectively located at 685,630,066 bp (SNP1) and 688,483,702 bp (SNP2). Notably, the physical distance between these two markers is 0.66 Mb on modern durum wheat and 2.85 Mb on wild emmer wheat, suggesting important genomic variations in this region between the two subspecies.

Twelve SNPs were isolated within the confidence interval around SNP1 and SNP2. The linkage disequilibrium analysis on these markers allowed us to determine the final size of the QTL on modern durum wheat and wild emmer wheat reference genomes, respectively, 3.6 and 3 Mb. According to the GrainGenes database, this region contains six already described QTLs, each related to six traits: phenolic acid content, semolina yellowness, number of seeds per plant, spike dry matter, number of spikes per plant, and leaf number per plant (Appendix 1: Table A1).

#### 3.1.2 An Inversion Covers the Entire QTL

**FIGURE A2:**
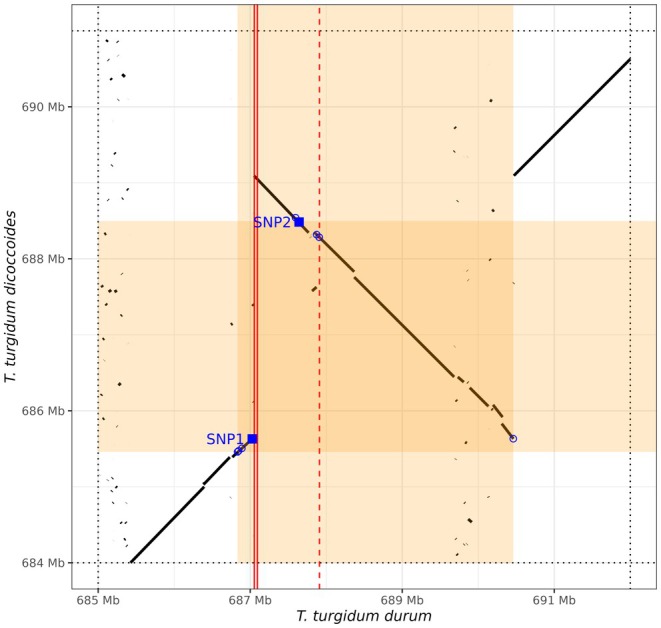
Dot plot of the genomic region containing the predation QTL (orange) on chromosome 2A between *T. turgidum* ssp. *durum* (Svevo v1) and *T. turgidum* ssp. *dicoccoides* (WEW v1). Blue circles represent the 12 within confidence interval markers position and the filled blue squares are the positions of SNP1 (AX‐89400647) and SNP2 (AX‐89632103). Vertical continuous and dashed red lines are respectively the positions of MYB transcription factors (the closest known gene from SNP1) and MYB protein.

The pairwise alignment of the QTL region between wild emmer wheat and modern durum wheat highlights a 3.4 Mb inversion between the two subspecies (Appendix 1: Figure A2). Within the QTL, 46 genes were detected on modern durum wheat and 34 on wild emmer wheat. On modern durum wheat, the closest downstream genes from SNP1 correspond to myeloblastosis (MYB) transcription factors (TdMYB2A033 and TdMYB2A034). On wild emmer wheat, because of the inversion, the MYB transcription factor gene is positioned downstream of SNP2.

### 3.2 Harvester Ant's Preferences for Spike Characteristics

#### 3.2.1 High Heritability on Spike Choice by Ants

The cafeteria experiment showed high heritability—that is, the proportion of phenotypic variation explained by genetic variation, for *M*_0_, as well as for predation variables *ML*_0–24_, *ML*_0–42_, *RML*_0–24_, *ML*_0–42_, and *RML*_0–42_. In contrast, heritability estimates for *ML*_24–42_ and *RML*_24–42_ were low (Table 1).

**TABLE 1:** Variance components and heritabilities of spike mass at *T*_0_ and predation variables, estimated from the cafeteria experiment. Data were collected from eight batches of 26 spikes, each batch being composed of 23 genotypes of EPO lines, two elite durum wheat genotypes, and one elite bread wheat genotype.

	*VG*	*VE*	*H* ^2^
*M*_0_	0.716	0.441	0.619
*ML*_0–24_	0.079	0.092	0.462
*ML*_24–42_	0.013	0.151	0.081
*ML*_0–42_	0.129	0.194	0.400
*RML*_0–24_	0.020	0.020	0.506
*RML*_24–42_	< 0.001	0.012	0.044
*RML*_0–42_	0.029	0.021	0.584

Abbreviations: *H* ^2^, broad sense individual heritability; *M*_0_, initial spike mass; *ML*, spike mass loss; *RML*, relative spike mass loss; *VE*, residual variance; *VG*, genotypic variance.

**FIGURE A3:**
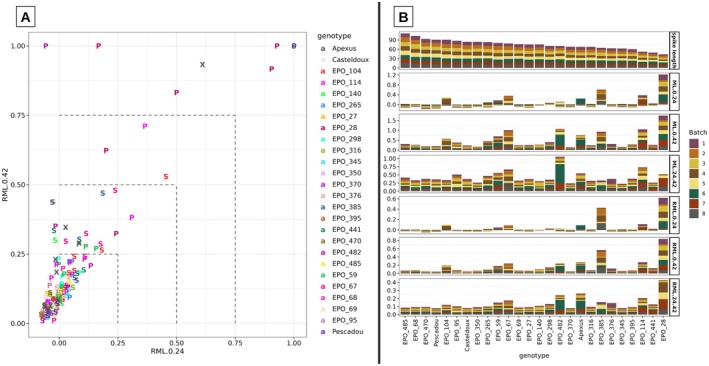
(A) Relative mass loss after 42 h depending on relative mass loss after 24 h. Each genotype is represented by a unique color. The letter P is the predated allele, S the safe allele and X the unknown one due to missing data in commercial lines; (B) Mean predation variables values for each predation variable for each genotype ordered by spike length. The color indicates the value for each batch.

Ants collected seeds from all batches. However, predation strongly varied among genotypes. After 24 h, eight spikes had lost more than 50% of their initial mass (four from EPO_28, three from EPO_385, and one from Apexus). By 42 h, an additional five spikes had also lost at least 50% of their initial weight or had disappeared entirely (two from EPO_28, one from EPO_482, one from EPO_114, and one from EPO_104) (Appendix 2: Figure A3). Notably, some spikes, particularly from genotype EPO_28, were entirely removed by ants that often buried them underground (Figure 3C). In contrast, spikes from other genotypes remained largely intact.

#### 3.2.2 Harvester Ants Prefer Short and Light Spikes

**FIGURE 5:**
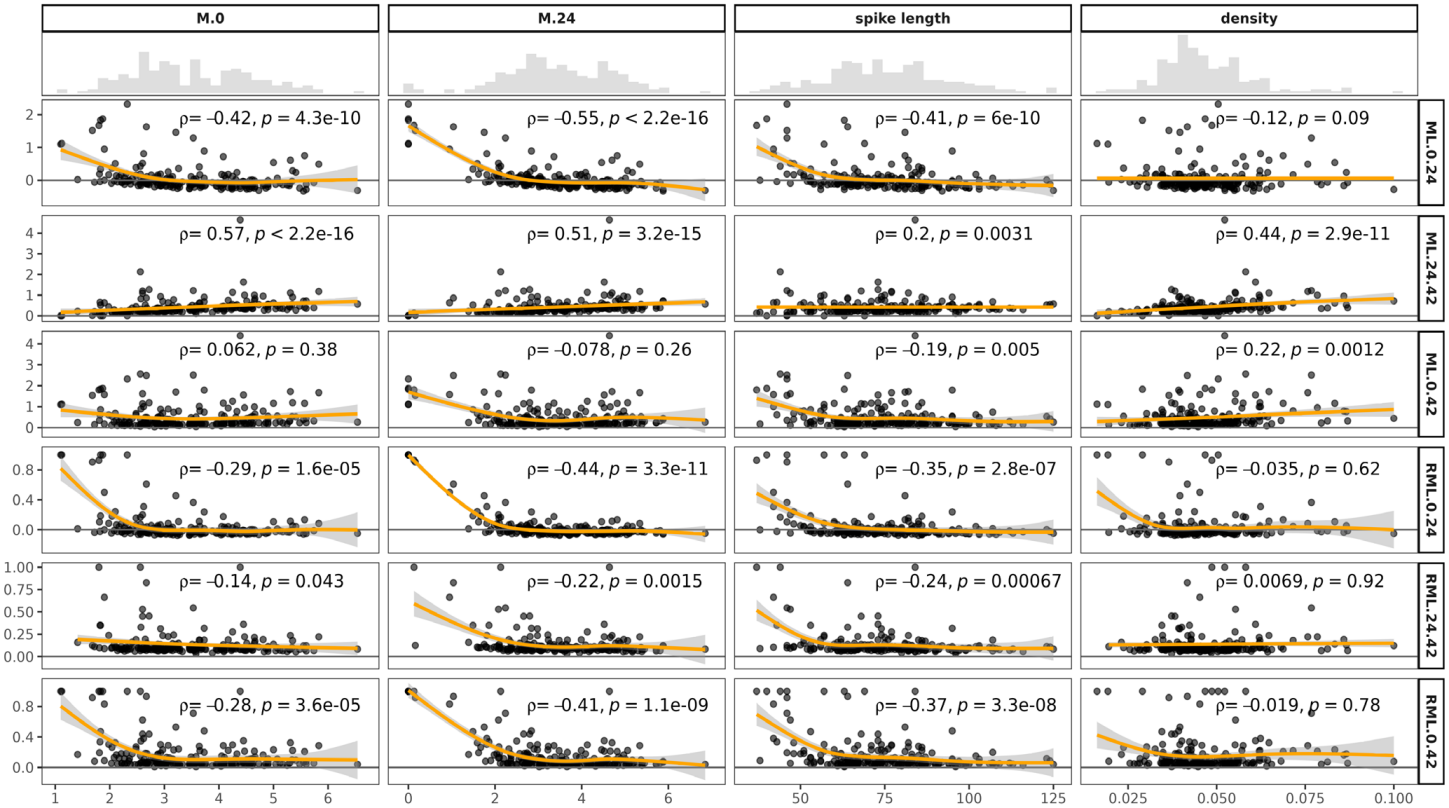
Scatter plot and spline of spike length, spike density, *M*_0_ and *M*_24_ in relation to the predation variables *ML* and *RML*. Spearman correlation test coefficients (rho) and *p* values are reported.

On average, absolute mass loss after 42 h (*ML*_0–42_) was 0.49 g per spike. *ML*_0–24_ was negatively correlated to *M*_0_ (rho = −0.42, *p* < 0.001) and spike length (rho = −0.41, *p* < 0.001), indicating that light and short spikes experienced larger mass loss during the first 24 h. *ML*_*24‐42*_ was positively correlated to *M*_24_ (rho = 0.51, *p* < 0.001), spike length (rho = 0.20, *p* = 0.003), and spike density (rho = 0.44, *p* < 0.001), indicating larger mass loss in heavier, longer, and denser spikes between 24 and 42 h. *ML*_0–42_ was negatively correlated to spike length (rho = −0.19, *p* = 0.004) and positively correlated to spike density (rho = 0.22, *p* = 0.001), indicating that mass loss was higher on shorter and denser spikes at the end of the experiment (Figure 5).

Average relative mass loss per spike after 42 h (*RML*_0–42_) was 16%. All spikes' relative mass losses were negatively correlated to *M*_0_ (*RML*_0–24_: rho = −0.29, *p* < 0.001; *RML*_24–42_: rho = −0.14, *p* = 0.04; *RML*_0–42_: rho = −0.28, *p* < 0.001) and to spike length (*RML*_0–24_: rho = −0.35, *p* < 0.001; *RML*_24–42_: rho = −0.24, *p* < 0.001; *RML*_0–42_: rho = −0.37, *p* < 0.001), indicating that ants still preferentially predated lighter and shorter spikes. Finally, no significant correlation was observed between *RML* and spike density (Figure 5).

#### 3.2.3 Allelic Variation Explains the Ant's Choice in the Cafeteria Experiment

**FIGURE 6:**
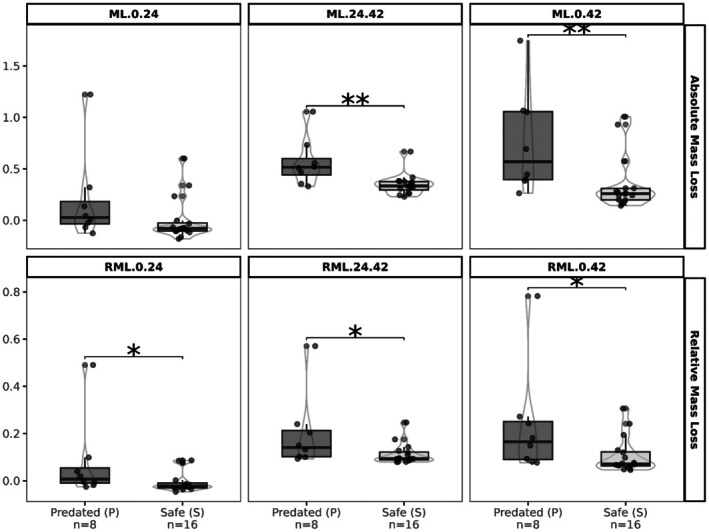
Effect of AX‐89400647 and AX‐89632103 variants on ant predation on 24 genotypes after 24, 42 h and from 24 to 42 h. In the GWAS analysis, the P variant is associated with higher ant preference.

Among the 26 genotypes of the cafeteria experiment, eight genotypes carried the predated allele (P) at both SNP1 and SNP2, 16 carried the “safe” allele (S) at both markers, and two had missing data in this region (Casteldoux and Apexus). Consequently, we only used the 24 genotypes with no missing data to test whether allelic variation at both loci explained variation in spike characteristics as well as variation in predation and ant preference observed in the cafeteria experiment. Allelic variation did not explain variation in spike characteristics (*M*_0_: *p* = 0.383, spike length: *p* = 1.00, spike density: *p* = 0.136). In contrast, it significantly explained part of the observed variation in ant preferences measured as *ML*_24–42_, *ML*_0–42_, *RML*_0–24_, *RML*_24–42_, and RML_0–42_: genotypes with P alleles were more predated in the cafeteria experiment than genotypes with S alleles (Figure 6). Only ML_0–24_ was not significantly associated with the allelic variant.

#### 3.2.4 No Correlation Between Ant Preferences and Seed Morphology Shift

**FIGURE 7:**
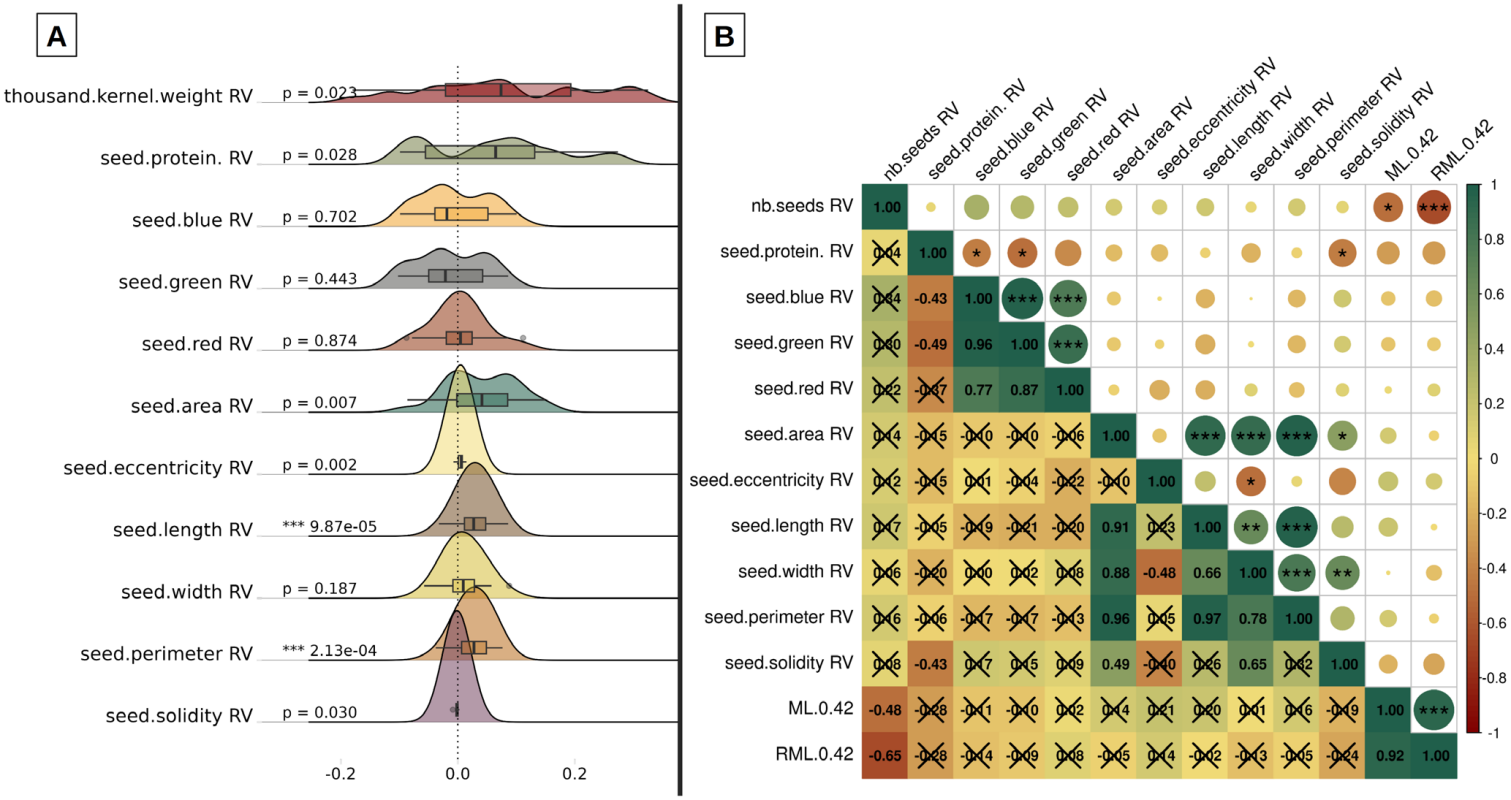
(A) Distribution of relative values (*RV*) for each seed trait measured with their Wilcoxon test *p* value. (B) Correlation matrix of the mean predation variables values per genotype and the relative values of each seed trait. Numerical values indicate the Spearman rho. Black crosses indicate nonsignificant tests and stars indicate the level of significativity (***P < 0.001, **P < 0.01, *P < 0.05).

Among the 208 spikes initially placed at nest entrances, 198 still had seeds after 42 h. When comparing characteristics of remaining seeds with those from controlled spikes, six out of 11 traits encompassed a significant increase (Figure 7): thousand seeds weight (*p* = 0.02), protein content (*p* = 0.02), perimeter (*p* < 0.001), length (*p* < 0.001), area (*p* = 0.007), and eccentricity (*p* = 0.002); solidity (*p* = 0.03) encompassed a significant decrease. Nevertheless, no significant correlation between the seeds' relative values (*RV*) and the mass loss variables after 42 h (*ML*_0–42_ and *RML*_0–42_) was established (Figure 7).

## 4 Discussion

Harvester ants have been studied in agricultural systems through several lenses, including ecosystem services (Evans and Gleeson 2016), soil structure and restoration (Evans et al. 2011; De Almeida et al. 2020), and—more rarely—direct predation on cultivated plants (Baraibar, Ledesma, et al. 2011). To our knowledge, our study is the first to explore interactions between harvester ants and cultivated crops through a genetic and evolutionary approach.

*Messor barbarus* ants exhibited a marked preference for specific wheat genotypes. Through a GWAS analysis, we identified a Quantitative Trait Loci (QTL) located at the distal end of the long arm of chromosome 2A, which is significantly associated with ant preference. This QTL explained up to 21% of the observed phenotypic variation in ant predation and was further validated via a cafeteria experiment, making it the first documented QTL in wheat associated with *M. barbarus* preference. While the genetic basis of wheat–insect interactions has already been described in the context of leaf herbivory by aphids (Smith et al. 2010; Batyrshina et al. 2020), our study provides evidence for a plant genetic effect on wheat spike predation by ants.

The QTL spans an entire chromosomal inversion between durum wheat and its wild progenitor. By limiting recombination, inversions have been shown to play a significant role in various evolutionary processes such as speciation, local adaptation, and the maintenance of complex phenotypes (Huang and Rieseberg 2020). Based on the currently available genomic data in durum wheat, we cannot assess whether this inversion is widespread among cultivated durum wheat varieties or represents a case of local adaptation specific to certain populations or elite varieties such as Svevo. Linking allelic frequency at this QTL locus and the occurrence of harvesting ants could help decipher the role of such structural variation in the evolution of wheat–ant interaction.

The genomic region we identified contains several candidate genes potentially involved in modulating ant attraction through their influence on seed cuticular and chemical traits. Among these are MYB transcription factors and MYB proteins, which are known to regulate the biosynthesis of key biochemical compounds in the plant cuticle and seed pericarp (N. Li et al. 2017; Flores et al. 2022), seed oil biosynthesis (Yang et al. 2025), and abiotic stress responses (Mao et al. 2011; Biswas et al. 2023)—or both (Bi et al. 2016; Luo et al. 2021). Previous studies have shown that the composition of oleic acids, cuticular waxes, and cuticular hydrocarbons plays a key role in ants' foraging decisions, as well as in nestmate recognition (Greene and Gordon 2003; Greene et al. 2013; Barbero 2016). This might suggest a direct impact of genotype chemical profiles on attractiveness to *Messor barbarus* . Apart from spike and seed morphology (Peleg et al. 2011; Shan and Osborne 2024), crop domestication has induced changes in their chemical compounds (Moreira et al. 2018; Chapuis et al. 2023). In this experiment, we did not measure seeds' chemical compounds. Investigating shifts in chemical signatures during domestication and breeding—particularly those known to be associated with ant attraction in wild species—would help decipher the role of chemical cues in plant–ant coevolution.

Harvester ants expressed marked preferences toward lighter and shorter spikes in the cafeteria experiment, suggesting that spike morphology plays a role in wheat‐ant interaction. On these traits, we observed the inverse effect of *ML*_24–42_, which may be due to a general loss of mass through drying in the sun, but also to the gradual depletion of resources. Moreover, during the cafeteria experiment, the ants still had access to wheat seeds from all genotypes that were not harvested yet. Thus, ants may have moved back to the preferred genotypes when done with the cafeteria experiment. As the small spikes were consumed first, the ants then turned to the larger ones. Relative mass loss (*RML*) therefore seems more relevant in explaining their preference. In line with these results, we found that the QTL locus explaining part of the variation in ant predation colocalizes with two published QTLs associated with spike morphology, namely the number of seeds per plant and spike dry matter. None of the traits we measured were associated with allelic variation at this QTL. The number of seeds and spike dry matter, which were not measured in our experiment, may thus represent promising candidate traits for future studies on crop‐ant interactions. To date, no other study has investigated the role of spike morphology on ant preferences. Few studies have reported that harvester ants prefer patches with high seed density (Heredia and Detrain 2005; García‐Meza et al. 2021), suggesting that they might optimize their foraging activities. Ant preference for smaller spikes in our study, which are likely easier to transport back to the nest, could fit with this hypothesis.

Our direct observations show that ants typically begin by removing the awns from the spikes, a behavior also described by (Lev‐Yadun et al. 2015). They then cut the glumes, palea, and lemma, subsequently either extracting the naked seeds directly or removing entire spikelets by cutting the nonbrittle rachis. A number of wild Poaceae species rely on topochory—the dispersal of seeds close to the mother plant—via trypanocarpy, described as a diaspore equipped with a hygroscopic drilling apparatus or a sharply pointed tip that enables it to penetrate the soil (Wood and Lenné 2018). Trypanocarpy has been documented in the wild progenitor of durum wheat (Elbaum et al. 2007). Awns thus play a functional role in seed dispersal. Harvester ants have specialized in dissecting the awns directly on the plant before they naturally fall to the ground and begin their dissemination phase (Schöning et al. 2004; Lev‐Yadun et al. 2015). This is also what we observed during our experiment: Ants climbed up the stems and began by removing all the awns before detaching the spikelets or directly opening the glumes to harvest the grains. When ants detach spikelets while the awns are still attached, the spikelet may be driven directly into the soil due to its straight trajectory—like an arrow. Additionally, if awns are still present and rainfall interrupts the harvest, those same seeds may bury themselves through hygroscopic drilling (Elbaum et al. 2008). As harvester ants prefer seeds without awns (Wandrag et al. 2021), removing awns may thus enhance their efficiency in seed collection. Modern durum wheat has shorter, thinner, and lighter awns compared to its wild progenitor (Peleg et al. 2010). Awn‐related traits may thus represent a promising avenue for investigating harvester ant behavior between modern and wild relative wheats.

In contrast to spike morphology, we did not find evidence that harvester ants select seeds according to their characteristics. As the change in seed morphology was not correlated with ant preferences, it might therefore be due to an uncontrolled experimental bias. Our results thus contrast with studies on nondomesticated species, where ants have been shown to choose larger and longer seeds (Heredia and Detrain 2005; Azcárate and Peco 2006). However, such studies often compare ant preferences between species displaying large interspecific seed size variation. Also, for some genotypes, we have observed that they tend to consume the entire spike. As seed mass and seed size not only vary between different genotypes but also within spikes (Baillot et al. 2018; Thakur et al. 2023), it seems unlikely that seed size is a major determinant of ants' choice in wheat. Furthermore, we did not find any relationship between ant preference and the protein content of the seed or the color of the seed. Cafeteria experiments with within‐spike seeds and between‐spike seeds of different sizes could help investigate this issue.

Our study demonstrates that allelic variation at a single locus influences ant foraging preferences among durum wheat inbred lines. These findings raise important questions regarding the origin of the allele associated with increased ant preference (referred to as P in our study). Modern agriculture, through the extensive use of chemical inputs for protection against pathogens and pests, might have indirectly counter‐selected natural plant defense mechanisms in favor of traits enhancing yield in seed crops (Whitehead et al. 2017) as a result of a trade‐off between growth and defense (Karasov et al. 2017; Giolai and Laine 2024). Thus, cultivated plants that once exhibited tolerance or even dispersal dependence to predators might have become largely dependent on human actions (Bernal and Medina 2018). Under this scenario, one might thus expect the frequency of allele P to have declined in the pool of elite durum wheat varieties and predation by ants to be rare in modern varieties. Testing this hypothesis would help decipher the driver of ant preferences during crop evolutionary history and if the low predation rate observed on modern crops by harvester ants (Baraibar, Ledesma, et al. 2011) is due to selection. Assessing genotype preferences of other *Messor* species may also help to know if allele P induces seed predation increase at the genus level. Exploring the relationship between the frequency of allele P and ant preferences across wild relatives of durum wheat in the Middle East may open the door to a better understanding of the evolutionary origin of this phenomenon, particularly given the fact that the *Messor* genus has transitioned toward seed‐specialization in this region (Juvé et al. 2025). In addition to providing us with a better understanding of the evolutionary history of crops, our findings call for investigating the interactions between plants and harvester ants in agrosystems other than intensive ones. For instance, crop diversification with the use of a broader range of genetic resources including wild species and landraces, and reduced inputs, as advocated for supporting the agroecological transition might affect plant–ant interactions and associated selective pressures on spike morphology. Finally, while it is well established that phenotypic changes in plants can significantly impact arthropod communities (Chen et al. 2018), it remains much less clear to what extent arthropods themselves have influenced trait evolution during the domestication process. Overall, our study calls for investigating more thoroughly whether ants have been forgotten witnesses or drivers of crop evolution.

## Author Contributions

**Clément Plessis:** conceptualization (lead), data curation (lead), formal analysis (lead), investigation (lead), methodology (lead), visualization (lead), writing – original draft (lead). **Aline Rocher:** investigation (supporting), resources (equal). **Frédéric Compan:** investigation (supporting), resources (equal). **Jonathan Romiguier:** conceptualization (supporting), writing – review and editing (supporting). **Jacques David:** conceptualization (supporting), formal analysis (supporting), funding acquisition (equal), resources (equal), supervision (equal), writing – original draft (supporting), writing – review and editing (supporting). **Hélène Fréville:** conceptualization (supporting), funding acquisition (equal), supervision (equal), visualization (supporting), writing – original draft (supporting), writing – review and editing (lead).

## Conflicts of Interest

The authors declare no conflicts of interest.

## Data Availability

All data and code used in this paper are available in the French Dataverse repository at: https://entrepot.recherche.data.gouv.fr/ (DOI: https://doi.org/10.57745/YX85WN).

## Appendix 1 Field Experiment

**TABLE A1:** Published QTL from grain genes which colocalized with the harvester ant predation QTL.

Trait	QTL name	DOI
Kernels per plant	QTL1742_2A‐Peng_et_al._2003	https://doi.org/10.1073/pnas.252763199
Leaf number	QTL1190_2A‐Giunta_et_al._2018	https://doi.org/10.3389/fpls.2018.00008
Spike dry weight	QTL1696_2A‐Peleg_et_al._2009b	https://doi.org/10.1111/j.1365‐3040.2009.01956.x
Phenolic acid content	QTL0842_PA‐Nigro_et_al__2017_	https://doi.org/10.1016/j.jcs.2017.01.022
Semolina color	QTL0953_2A‐Blanco_et_al._2011_	https://doi.org/10.1016/j.jcs.2011.07.002
Spike per plant	QTL1191_2A‐Giunta_et_al._2018	https://doi.org/10.3389/fpls.2018.00008
